# Prognostic value of triglyceride glucose (TyG) index in patients with acute decompensated heart failure

**DOI:** 10.1186/s12933-022-01507-7

**Published:** 2022-05-31

**Authors:** Rong Huang, Ziyan Wang, Jianzhou Chen, Xue Bao, Nanjiao Xu, Simin Guo, Rong Gu, Weimin Wang, Zhonghai Wei, Lian Wang

**Affiliations:** 1 Department of Cardiology, Nanjing Drum Tower Hospital, The Affiliated Hospital of Nanjing University Medical School, Nanjing, 210008 Jiangsu China; 2 Department of Cardiology, Nanjing Drum Tower Hospital Clinical College of Jiangsu University, Nanjing, 210008 Jiangsu China; 3 Department of Endocrinology, Nanjing Drum Tower Hospital, The Affiliated Hospital of Nanjing University Medical School, Nanjing, 210008 Jiangsu China

**Keywords:** Triglyceride glucose index, Acute decompensated heart failure, All-cause death, Cardiovascular death, Major adverse cardiac and cerebral events

## Abstract

**Background:**

The triglyceride glucose (TyG) index has been proposed as a reliable marker of insulin resistance (IR) and an independent predictor of cardiovascular disease risk. However, its prognostic value in patients with acute decompensated heart failure (ADHF) remains unclear.

**Methods:**

A total of 932 hospitalized patients with ADHF from January 1st, 2018 to February 1st, 2021 were included in this retrospective study. The TyG index was calculated as ln [fasting triglyceride level (mg/dL) × fasting plasma glucose level (mg/dL)/2]. Patients were divided into tertiles according to TyG index values. The primary endpoints were all-cause death, cardiovascular (CV) death and major adverse cardiac and cerebral events (MACCEs) during follow-up. We used multivariate adjusted Cox proportional hazard models and restricted cubic spline analysis to investigate the associations of the TyG index with primary endpoints.

**Results:**

During a median follow-up time of 478 days, all-cause death, CV death and MACCEs occurred in 140 (15.0%), 103 (11.1%) and 443 (47.9%) cases, respectively. In multivariate Cox proportional hazard models, the risk of incident primary endpoints was associated with the highest TyG tertile. After adjustment for confounding factors, hazard ratios (HRs) for the highest tertile (TyG index ≥ 9.32) versus the lowest tertile (TyG index < 8.83) were 2.09 (95% confidence interval [CI], 1.23–3.55; p = 0.006) for all-cause death, 2.31 (95% CI, 1.26–4.24; p = 0.007) for CV death and 1.83 (95% CI, 1.18–3.01; p = 0.006) for MACCEs. Restricted cubic spline analysis also showed that the cumulative risk of primary endpoints increased as TyG index increased. When the TyG index was used as a continuous variable, the hazard ratios of the three primary endpoints rapidly increased within the higher range of the TyG index (all cause death, TyG > 9.08; CV death, TyG > 9.46; MACCEs, TyG > 9.87).

**Conclusions:**

The elevated TyG index was independently associated with poor prognosis, and thus would be useful in the risk stratification in patients with ADHF.

## Introduction

Despite similar clinical presentations, acute decompensated heart failure (ADHF) is a highly heterogeneous syndrome with incompletely understood pathophysiology [1, 2]. In contrast to increasing therapeutic options of chronic heart failure (CHF), no new drug for ADHF has been approved in decades [2, 3]. Considering higher rates of morbidity and mortality in ADHF patients than in those with recently diagnosed HF, it is critical to identify high-risk groups before discharge to improve their prognosis [4, 5]. Previous studies have showed some clinical biomarkers, such as elevated BNP, hyponatremia, anemia, and worsening renal function, are associated with poor outcomes in ADHF [6–9]. However, there are few well-validated biomarkers and effective clinical tools in patients with ADHF [2]. Therefore, appropriate risk stratification of ADHF and further individualization of care needs to be further explored [2, 10].

Insulin resistance (IR), a marker of metabolic disorders and systemic inflammation, is an independent and significant risk factor for HF and cardiovascular (CV) death [11–13]. The homeostasis model assessment (HOMA) has been used as a relatively simple and reliable method for assessing IR in previous studies requiring fasting insulin and glucose [14, 15]. The triglyceride-glucose (TyG) index, combined fasting plasma glucose (FPG) and triglyceride levels (TGs), was firstly proposed by Unger G et al. in 2013 as an alternative indicator of IR [16]. Many studies have demonstrated that the TyG index outperformed the HOMA in assessing IR [17–19]. A large number of studies have indicated that the TyG index is positively correlated with the incidence rates of carotid artery atherosclerosis, coronary artery disease, hypertension, myocardial infarction and other cardiovascular diseases [20–24]. Meanwhile, the TyG index is also a reliable and convenient predictor of adverse prognosis in patients with cardiovascular disease [25–27].

However, there are limited clinical studies assessing the TyG index in ADHF. Therefore, we conducted a retrospective cohort study to investigate the relationship between the TyG index and the prognosis in ADHF patients.

## Materials and methods

### Study population

**Fig. 1:**
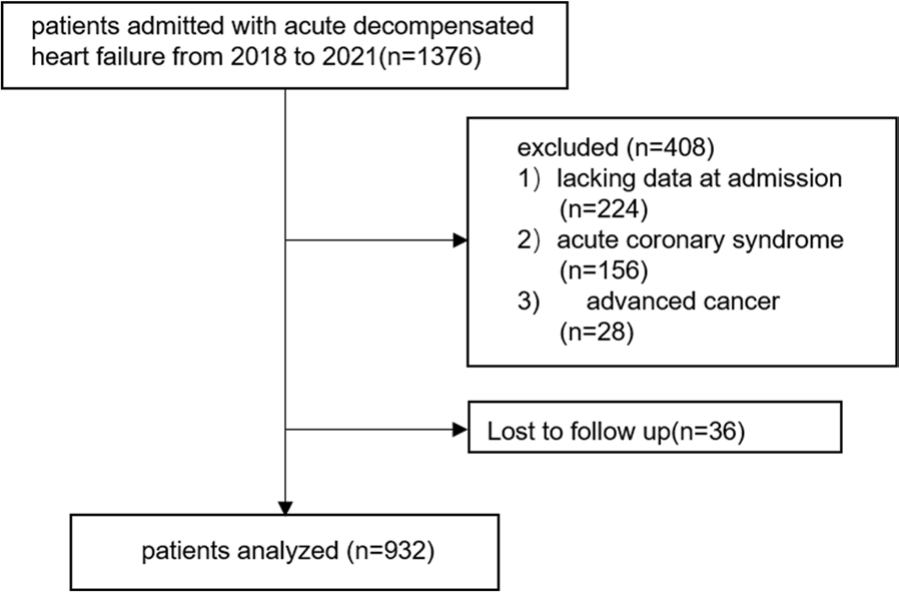
Flow diagram of patient selection

This is a single-center retrospective analysis of 1376 consecutive ADHF patients admitted to our hospital from January 1st, 2018 to February 1st, 2021. ADHF was defined according to the definitions established in the 2021 ESC Guidelines for the diagnosis and treatment of acute and chronic heart failure [28]. Of the 1376 patients, 444 were excluded for meeting the exclusion criteria, i.e., (1) lack of data at admission, (2) acute coronary syndrome, (3) advanced cancer, (4) lost to follow up. Finally, 932 patients were included in this study (Fig. 1). This retrospective study was performed in line with the Declaration of Helsinki, with the approval from the ethics committee of Nanjing Drum Tower Hospital.

### Data collection and definitions

Patient demographics, medical history, laboratory test results, echocardiographic data and medications at admission were collected from the electronic medical recording system by trained physicians. The first group of peripheral venous blood samples was collected after overnight fasting (> 8 h) and was measured in the laboratory department. Body mass index (BMI) was defined as weight (kg)/ (height [m]^2^). Hypertension was defined as systolic blood pressure (SBP) ≥ 140 mmHg and/or diastolic blood pressure (DBP) ≥ 90 mmHg, any use of the antihypertensive drugs, or a having a history of hypertension. Diabetes mellitus (DM) was defined as fasting plasma glucose (FPG) ≥ 126 mg/dL or hemoglobin A1c (HbA1c) ≥ 6.5%, or a self-reported history of diabetes [29]. Hyperlipidemia was defined as fasting total cholesterol (TC) ≥ 240 mg/dL, low-density lipoprotein cholesterol (LDL-C) > 160 mg/dL, TGs ≥ 150 mg/dL, high-density lipoprotein cholesterol (HDL-C) < 40 mg/dL, or previous use of lipid-lowering drugs.

Hemoglobin, hematocrit, uric acid, red blood cell distribution width (RDW), FPG, HbA1c, total cholesterol, triglycerides, LDL-C, HDL-C, creatinine, C-reactive protein (CRP), B-type natriuretic peptide (BNP), white blood cell count (WBC), serum sodium, serum potassium, albumin (ALB), alanine aminotransferase (ALT) and aspartate transaminase (AST) were measured.

### TyG calculation

$${\text{TyG }} = {\text{ ln }}\left[ {{\text{fasting triglyceride }}\left( {{\text{mg}}/{\text{dL}}} \right) \, \times  {\text{fasting plasma glucose }}\left( {{\text{mg}}/{\text{dL}}} \right)/{2}} \right]$$

### Endpoints and follow-up

Three predefined primary endpoints were examined, including (1) all-cause death, defined as CV death or non-CV death; (2) CV death, defined as fatal stroke and myocardial infarction (MI), congestive heart failure, malignant arrhythmia, or other structural or functional cardiac diseases; (3) major adverse cardiac and cerebral events (MACCEs), defined as non-fatal MI, non-fatal stroke, or worsening of heart failure. Non-fatal stroke included ischemic and hemorrhagic strokes.

Patients were followed up by telephone and/or clinic visits every six months by well-trained doctors.

### Statistical analysis

Continuous variables were expressed as mean ± standard deviation (SD) and median [interquartile range (IQR)] for those with normal and skewed distributions, respectively. Categorical variables were expressed as number (percentage).

The patients were divided into tertiles according to their TyG index levels: tertile 1 (T1), TyG index < 8.83; tetile 2 (T2), TyG index ≥ 8.83 and < 9.32; tertile 3 (T3), TyG index ≥ 9.32. Continuous variables were compared by analysis of variance (ANOVA) or the Kruskal–Wallis test among the three groups. Categorical variables were compared by the χ^2^ test among groups.

The cumulative event-free survival rates of the three endpoints were analyzed using the Kaplan–Meier plots and the log-rank test. Additionally, to rule out the effects of confounding factors, adjusted Kaplan–Meier survival curves were generated. Adjusted survival curves were obtained using non-parametric estimated weights [30–32]. Multivariate Cox proportional hazards models were applied to test the associations of the TyG index with the incidence rates of the three primary outcomes. Visualization of Schoenfeld residuals were used to validate the assumptions of proportional hazards before these analyses. The baseline variables with p < 0.1 or clinically significant were selected and included in the Cox proportional hazards models. Multicollinearity was tested in the multivariate models with a threshold of variance inflation factor < 4. Finally, four multivariable regression models were remained: Model 1, adjustment for age, sex, BMI, systolic blood pressure, diastolic blood pressure and heart rate; Model 2, adjustment for variables included in Model 1 plus HbA1C, C-creative protein, hematocrit, red blood cell distribution width, BNP, sodium, albumin, creatinine, uric acid, ALB, HDL-C and LVEF; Model 3, adjustment for variables included in Model 2 plus history of hypertension, ischemic cardiomyopathy, diabetes mellitus, valvular heart disease, atrial fibrillation and hyperlipidemia; Model 4, adjustment for variables included in Model 3 plus statin, aldosterone antagonist, digoxin, diuretic, β-lockers, antiplatelet agent and/or angiotensin-converting enzyme inhibitor (ACEI)/angiotensin receptor inhibitors (ARB)/angiotensin receptor neprilysin inhibitor (ARNI), insulin, sodium-glucose contrasporter-2 inhibitor (SGLT2i), and metformin uses. Hazard ratios (HRs) were calculated, and the results were reported as HRs and 95% confidence intervals (CIs). The lowest tertile of the TyG index was used as a reference in the four models. A restricted cubic spline analysis was performed to reflect the dose–response relationship between the TyG index and the risk of the three primary outcomes. We also conducted subgroup analyses, including age (< 70 versus ≥ 70 years), sex (male versus female), BMI (< 24 versus ≥ 24 kg/m^2^), and history of diabetes mellitus (no versus yes). Interactions between subgroups were tested by the likelihood ratio test. All data were analyzed with R version 4.1.0 and SPSS for windows version 26, and p < 0.05 was considered to indicate statistical significance.

## Results

### General patient characteristics

The baseline clinical characteristics of the included patients by TyG tertile are presented in Table 1. The median follow-up time was 478 days. The mean age of the participants was 69 years, and 37.9% participants were female. The proportions of individuals with previous hypertension, ischemic cardiomyopathy (IMD), DM and hyperlipidemia were significantly higher in the highest TyG index tertile, as well as BMI, FPG, HbA1C, CRP, UA, hematocrit, WBC, ALB, total cholesterol, triglyceride, LDL-C and hemoglobin levels; antiplatelet agent, statin, insulin, metformin, sodium-glucose cotransporter 2 inhibitor (SGLT2i) and other hypoglycemic drugs uses were also enhanced (all p < 0.05). Meanwhile, the highest TyG index tertile had younger age; lower HDL-C, RDW, sodium, left atrial diameter (LAD) and LVEF, and lower proportions of valvular heart disease, atrial fibrillation and anemia (all p < 0.05).

**Table 1:** Baseline Clinical Characteristics by TyG Group

Characteristic	Total	Tertile I (Lowest) (n = 310) TyG < 8.83	Tertile II (Median) (n = 309) 8.83 ≤ TyG < 9.32	Tertile III (Highest) (n = 313) TyG ≥ 9.32	p Value	
Female, n (%)	353(37.9)	95(26.9)	133(37.7)	125(35.4)	< 0.001	##
Age (years)	70(61,80)	73(64,82)	71(63,79)	67(56,76)	< 0.001	#,***,&&
BMI (kg/m2)	24.2(21.6,27.1)	23.3(20.5,26.1)	24.1(21.5,26.5)	25.2(22.7,28.1)	< 0.001	#,***,&&
Heart Rate (bpm)	81(70,97)	80(68,95)	81(70,97)	84(71,98)	0.261	
SBP (mmHg)	129(114,145)	127(114,147)	128(113,142)	130(116,144)	0.456	
DBP (mmHg)	75(66,87)	75(65,85)	75(66,87)	76(67,87)	0.507	
Medical history, n (%)						
Ischemic cardiomyopathy	347(37.2)	105(33.9)	99(32)	143(45.7)	0.001	*,&&
None-ischemic cardiomyopathy	197(21.1)	53(17.1)	74(23.9)	70(22.4)	0.091	
Valvular heart disease	174(18.7)	70(22.6)	60(19.4)	44(14.1)	0.022	*
Atrial fibrillation	404(43.3)	169(54.5)	139(45.0)	96(30.7)	< 0.001	***,&&&
Hypertension	563(60.4)	172(55.5)	186(60.2)	205(65.5)	0.038	*
Hyperlipidemia	72(43.3)	5(1.6)	20(6.5)	47(15.0)	< 0.001	#,***,&&
Diabetes mellitus	306(32.8)	64(20.6)	83(26.9)	159(50.8)	< 0.001	***,&&&
Anemia	429(46.0)	177(57.3)	134(44.1)	118(37.9)	< 0.001	##,*
Hemoglobin(g/L)	128.0(110.1,142.4)	125.0(107.6,138.1)	129.5(111.3,141.8)	132.0(114.0,145.0)	< 0.001	***
FPG (mg/dL)	91.8(81.9,112.0)	82.6(75.6,91.1)	92.2(83.9,104.9)	118.44(92.2,151.7)	< 0.001	###,***,&&&
HbA1c (%)	6.1(5.6,7.0)	6.0(5.6,6.4)	6.1(5.6,6.8)	6.8(5.8,7.9)	< 0.001	***,&&&
BNP (pg/mL)	589.5(307.8,1230.0)	591.5(339.3,1167.5)	606(123.5,1088.5)	552.0(177.3,926.8)	0.476	
CRP (mg/L)	4.5(2.9,12.4))	4.4(2.9,9.3)	4.6(3.0,14.7)	4.4(3.0,14.5)	0.330	
Hematocrit (%)	38.4(33.8,42.2)	37.3(33.1,41.1)	38.7(34.1,42.1)	39.10(35.1,43.2)	0.001	***
ALB (g/L)	38.2(35.4,40.5)	37.4(35.1,39.2)	38.4(36.0,40.8)	39.1(35.5,41.4)	< 0.001	#,***
UA (μmol/L)	439.0(343.25,550.75)	419.5(328.3,528.3)	430.0(325.0,558.5)	448(372.5,555.0)	0.033	*
Creatinine (μmol/L)	84.3(67.0,113.0)	84.4(69.1,107.1)	87.0(65.3,113.0)	85.10(65.7,119.8)	0.971	
Sodium (mmol/L)	138.3(135.5,141.5)	138.3(135.4,141.4)	139.0(135.8,141.8)	138.2(135.2,141.2)	0.458	
Potassium (mmol/L)	3.9(3.6,4.2)	3.9(3.5,4.2)	3.9(3.6,4.2)	3.90(3.6,4.3)	0.707	
WBC (10^9/L)	6.2(5.0,7.8)	5.5(4.5,6.9)	6.1(5.1,7.7)	6.95(5.7,8.7)	< 0.001	###,***,&&&
RDW (%)	13.7(12.9,13.7)	14.1(13.2,15.1)	13.6(12.9,14.7)	13.4(12.8,14.3)	< 0.001	***,&
Total cholesterol (mg/dL)	138.16(114.07,170.15)	120.36(103.93,145.11)	141.26(118.71,168.60)	156.74(129.93,188.90)	< 0.001	###,***,&&&
Triglyceride (mg/dL)	90.27(29.00,54.91)	61.07(22.43,30.55)	92.04(35.19,46.40)	149.57(50.08,83.91)	< 0.001	###,***,&&&
HDL-C (mg/dL)	37.15(29.78,46.79)	40.25(33.06,50.46)	38.70(30.55,49.50)	33.28(27.07,40.60)	< 0.001	###,***
LDL-C (mg/dL)	77.79(58.05,101.32)	65.79(50.46,82.75)	79.72(60.71,103.25)	89.78(65.74,116.01)	< 0.001	###,***,&&
AST (U/L)	23.0(17.3,33.6)	23.1(17.5,32.8)	24.0(17.5,33.0)	21.60(17.1,35.4)	0.568	
ALT (U/L)	19.4(12.9,32.5)	18.6(11.9,28.2)	19.5(13.1,31.9)	20.60(14.1,38.9)	0.027	
IVSTD (cm)	0.9(0.8,1.0)	0.9(0.8,1.0)	0.90(0.8,1.0)	0.90(0.8,1.1)	0.357	
LVPWTD (cm)	0.9(0.8,1.0)	0.90(0.8,1.0)	0.90(0.8,1.0)	0.90(0.8,1.0)	0.173	
LVDD (cm)	5.9(5.2,6.5)	5.8(5.1,6.5)	5.9(5.2,6.5)	5.9(5.2,6.7)	0.270	
LAD (cm)	4.8(4.4,5.3)	5.0(4.5,5.5)	4.8(4.5,5.3)	4.7(4.2,5.2)	< 0.001	#,***,&
LVEF, n (%)					0.048	*
≤ 40%	493(52.9)	144(46.5)	168(54.4)	181(57.8)	-	
41% ~ 49%	154(16.5)	63(20.3)	46(14.9)	45(14.4)	-	
≤ 50%	285(30.6)	103(33.2)	95(30.7)	87(27.8)	-	
Medications at admission, n (%)						
Antiplatelet agent	412(44.2)	116(37.4)	135(43.7)	161(51.4)	0.002	**
ACEI/ARB/ARNI	150(16.1)	52(16.8)	43(13.9)	55(17.6)	0.428	
Beta-blocker	726(77.9)	236(76.1)	242(78.3)	248(79.2)	0.633	
Statins	509(54.6)	154(49.7)	164(53.1)	191(61.0)	0.014	*
Diuretics	819(87.9)	270(87.1)	281(90.9)	268(53.7)	0.103	
Loop	810(86.9)	268(86.5)	279(90.0)	263(84.0)	0.072	
Thiazide	51(5.5)	19(6.1)	15(4.9)	17(5.4)	0.784	
Digoxin	102(10.9)	38(12.3)	24(7.8)	40(12.8)	0.079	
Aldosterone antagonist	375(40.2)	128(41.3)	128(41.4)	119(38.0)	0.616	
Insulin	211(22.6)	41(13.2)	58(18.8)	112(35.8)	< 0.001	**&&
Metformin	106(11.4)	15(4.8)	32(10.4)	59(18.8)	< 0.001	##,***&&
Sulfonylureas	4(0.4)	1(0.1)	1(0.1)	2(0.2)	0.785	
SGLT2i	27(2.9)	5(1.6)	5(1.6)	17(1.8)	0.005	**,&&
Other hypoglycemic drugs	172(18.5)	34(3.6)	43(4.6)	95(10.2)	< 0.001	***,&&

*BMI* body mass index, *SBP* systolic blood pressure, *DBP* diastolic blood pressure, *FPG* fasting plasma glucose, *BNP* B-type natriuretic peptide, *CRP* C-creative protein, *ALB* albumin, *UA* uric acid, *Cr* creatinine; *Na* sodium; *K* potassium, *WBC* white blood cell, *RDW* red blood cell distribution width, *HDL-C* high-density lipoprotein cholesterol, *LDL-C* low-density lipoprotein-C, *AST* alanine aminotransferase, *ALT* aspartate transaminase; *IVSTD* interventricular septal thickness at diastole, *LVPWTD* left ventricular posterior wall end-diastolic thickness, *LVDD* left ventricular end-diastolic diameter, *LAD* left atrial diameter, *LVEF* left ventricular ejection fraction, *ACEI* angiotensin-converting enzyme inhibitor, *ARB* angiotensin receptor inhibitor, *ARNI* angiotensin receptor neprilysin inhibitor, *SGLT2i* Sodium-glucose cotransporter 2 inhibitor

^#^P < 0.05, ##P < 0.01, ### P < 0.001: T1 group vs T2 group

^*^P < 0.05, **P < 0.01, *** P < 0.001: T1 group vs T3 group

^&^P < 0.05, &&P < 0.01, &&& P < 0.001: T2 group vs T3 group

### Predictive ability of the TyG index for the primary outcomes

During the follow-up period, all-cause death was found in 140 (15.0%) cases, CV death occurred in 103 (11.1%) cases and MACCEs were detected in 443 (47.9%) cases. The patients who died included 45 (14.5%) in the lowest tertile, 42 (13.6%) in the median tertile and 53 (16.9%) in the highest tertile. The patients who died from cardiovascular events included 34 (11%) in the lowest tertile, 28 (9.1%) in the median tertile and 41 (13.1%) in the highest tertile. Individuals with major adverse cardiac and cerebral events included 156 (51.1%) in the lowest tertile, 130 (42.3%) in the median tertile and 157 (50.2%) in the highest tertile.

**Fig. 2:**
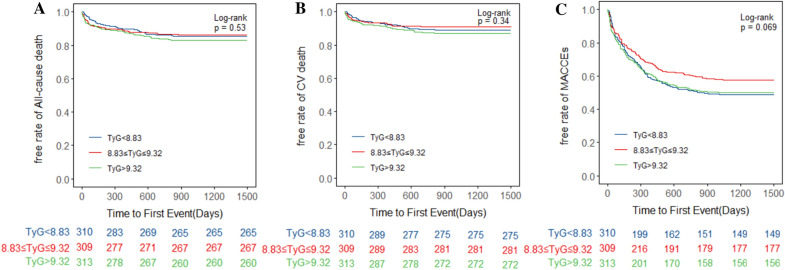
Kaplan–Meier analysis of **A** All-cause death, **B** CV death and **C** MACCEs in various TyG groups. CV, cardiovascular; MACCEs, major adverse cardiac and cerebral events; TyG, triglyceride glucose index

**Fig. 3:**
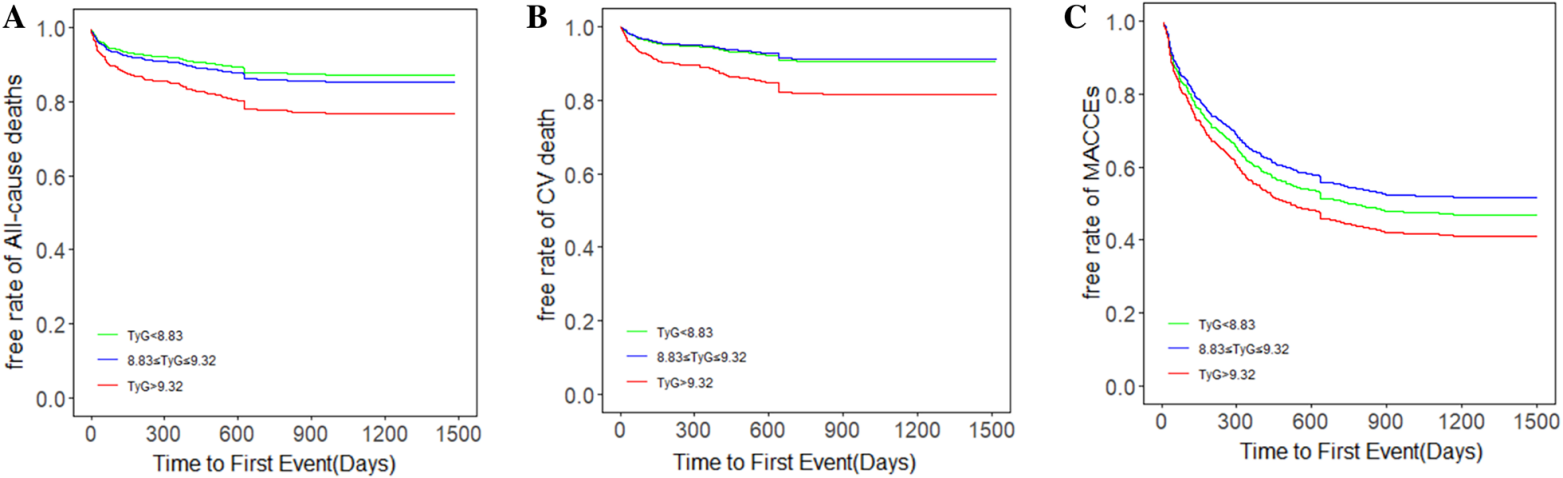
Adjusted survival curves for **A** All-cause death, **B** CV death and **C** MACCEs in different TyG groups after adjustment for age, sex, TyG index, body mass index, systolic blood pressure, diastolic blood pressure, heart rate, HbA1C, C-creative protein, hematocrit, red blood cell distribution width, BNP, sodium, albumin, creatinine, uric acid, ALB, HDL-C, LVEF, history of hypertension, ischemic cardiomyopathy, diabetes mellitus, valvular heart disease, atrial fibrillation and hyperlipidemia, and statin, aldosterone antagonist, digoxin, diuretic, β-blocker, antiplatelet agent, ACEI/ARB/ARNI, insulin, sodium-glucose contrasporter-2 inhibitor, and metformin uses. CV, cardiovascular; MACCEs, major adverse cardiac and cerebral events; TyG, triglyceride glucose index

The unadjusted Kaplan–Meier curves demonstrated no significant differences among tertiles in the three predefined primary endpoints (Fig. 2). However, after adjusting for variables in Model 4, we draw the adjusted survival curves, which showed marked differences among the three TyG groups and three endpoints (Fig. 3). Table 2 showed the four multivariate Cox proportional hazards models utilized to test the correlations between the TyG index and primary outcomes. Four Cox proportional hazards models were established for sensitivity analysis. Consequently, the highest tertile of the TyG index was associated with higher incidence of all-cause death and cardiovascular death (all-cause death, adjusted HR = 2.09, 95% CI 1.23–3.55, p = 0.006; cardiovascular death, adjusted HR = 2.31, 95% CI 1.26–4.24, p = 0.007). Meanwhile, there were no significant differences between the lowest and median tertile groups. The incidence of MACCEs was significantly different among tertiles in Model 2, Model 3 and Model 4 (Model 2, T2 adjusted HR = 1.65, 95% CI 1.18–2.30, p = 0.003; T3 adjusted HR = 1.71, 95% CI 1.15–2.58, p = 0.008; Model 3, T2 adjusted HR = 1.64, 95% CI 1.17–2.31, p = 0.004; T3 adjusted HR = 1.72, 95% CI 1.13–2.62, p = 0.012; Model 4, T2 adjusted HR = 1.57, 95% CI 1.11–2.78, p = 0.023; T3 adjusted HR = 1.83, 95% CI 1.18–3.01, p = 0.006), while no significant differences were observed in Model 1.

**Table 2:** HR (95% CI) of primary outcomes according to TyG index in the four Models

			Model1		Model2		Model3		Model4	
Outcomes	TyG groups	Events, n(%)	HR (95% CI)	p Value	HR (95% CI)	p Value	HR (95% CI)	p Value	HR (95% CI)	p Value
All-cause death	T1	45(14.5)	Ref.		Ref.		Ref.		Ref.	
	T2	42(13.6)	1.11(0.73–1.69)	0.628	1.04(0.67–1.63)	0.848	1.07(0.68–1.67)	0.765	1.07(0.67–1.1.74)	0.761
	T3	53(16.9)	1.70(1.13–2.57)	0.011	1.75(1.08–2.83)	0.023	1.83(1.12–3.01)	0.015	2.09(1.23–3.55)	0.006
CV death	T1	34(11.0)	Ref.		Ref.		Ref.		Ref.	
	T2	28(9.1)	0.97(0.59–1.60)	0.918	0.93(0.55–1.58)	0.801	0.91(0.53–1.56)	0.736	0.93(0.52–1.65)	0.802
	T3	41(13.1)	1.61(1.01–2.58)	0.046	1.89(1.08–3.28)	0.025	1.91(1.08–3.37)	0.026	2.31(1.26–4.24)	0.007
MACCEs	T1	156(51.1)	Ref.		Ref.		Ref.		Ref.	
	T2	130(42.3)	1.21(0.91–1.60)	0.186	1.65(1.18–2.30)	0.003	1.64(1.17–2.31)	0.004	1.57(1.11–2.78)	0.023
	T3	157(50.2)	1.32(0.96–1.83)	0.083	1.71(1.15–2.58)	0.008	1.72(1.13–2.62)	0.012	1.83(1.18–3.01)	0.006

*CI* confidence interval, *TyG* triglyceride glucose index, *HR* hazard ratio

Model 1: adjusted for gender, age, body mass index, systolic blood pressure, diastolic blood pressure and heart rate;

Model 2: adjusted for Model 1 + HbA1C, C-creative protein, hematocrit, red blood cell distribution width, BNP, sodium, albumin, creatinine, uric acid, ALB, HDL-C and LVEF;

Model 3: adjusted for Model 2 + history of hypertension, ischemic cardiomyopathy, diabetes mellitus, valvular heart disease, atrial fibrillation and hyperlipidemia;

Model 4: adjusted for Model 3 + use of statins, aldosterone antagonist, digoxin, diuretics, β-Blockers, antiplatelet agent, ACEI/ARB/ARNI, insulin, sodium-glucose contrasporter-2 inhibitor, and metformin

### The TyG index as a continuous variable

**Fig. 4:**
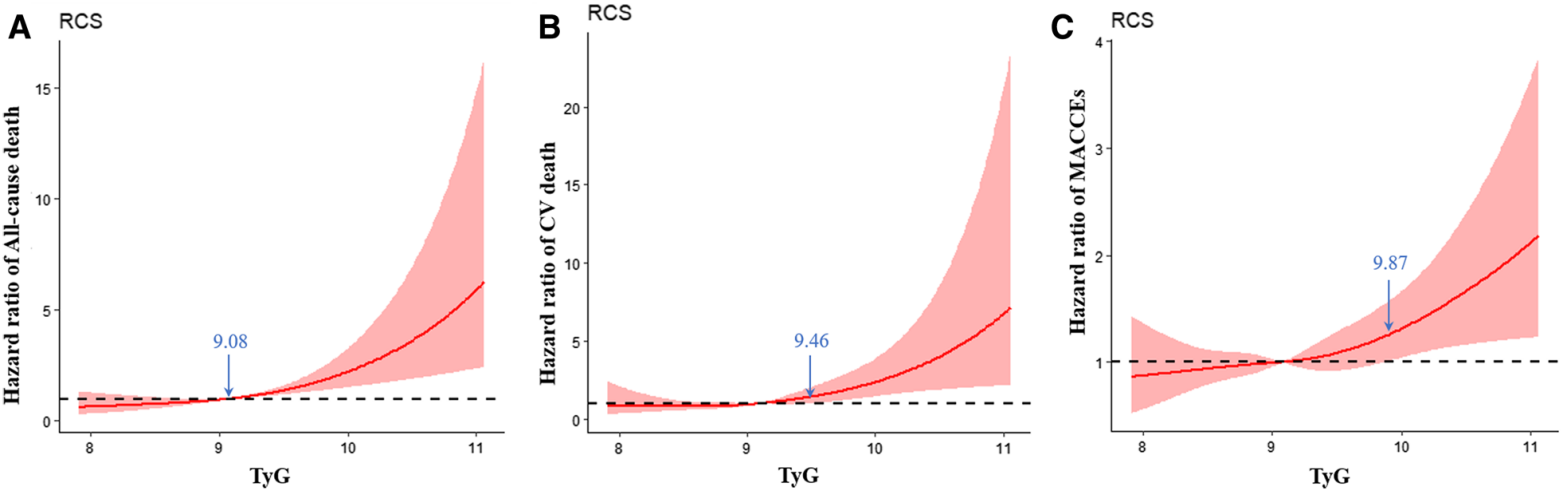
Multivariable-adjusted hazard ratios for **A** All-cause death, **B** CV death and **C** MACCEs based on restricted cubic spines for the TyG index. Red lines represented references for hazard ratios, and red areas represent 95% confidence intervals. The model was adjusted for age, sex, TyG index, body mass index, systolic blood pressure, diastolic blood pressure, heart rate, HbA1C, C-creative protein, hematocrit, red blood cell distribution width, BNP, sodium, albumin, creatinine, uric acid, ALB, HDL-C, LVEF, history of hypertension, ischemic cardiomyopathy, diabetes mellitus, valvular heart disease, atrial fibrillation, hyperlipidemia, and statin, aldosterone antagonist, digoxin, diuretic, β-blocker, antiplatelet agent, ACEI/ARB/ARNI, insulin, sodium-glucose contrasporter-2 inhibitor, and metformin uses

In Fig. 4, the restricted cubic spline models showed that the risk of the three endpoints was initially flat and then rapidly increased. The risk of all-cause mortality increased rapidly for TyG index above 9.08 (HR per SD increase = 1.48, 95% CI 1.24–1.76, p for non-linearity = 0.001). For TyG index above 9.46, the hazard ratio of CV death per standard deviation of the TyG index was 2.06, (95%CI 1.31–3.21, p for non-linearity = 0.003). The turning point of the TyG index was 9.87 for the risk of MACCEs (HR per SD increase = 1.56, 95% CI 1.14–2.12, p for non-linearity = 0.034).

**Fig. 5:**
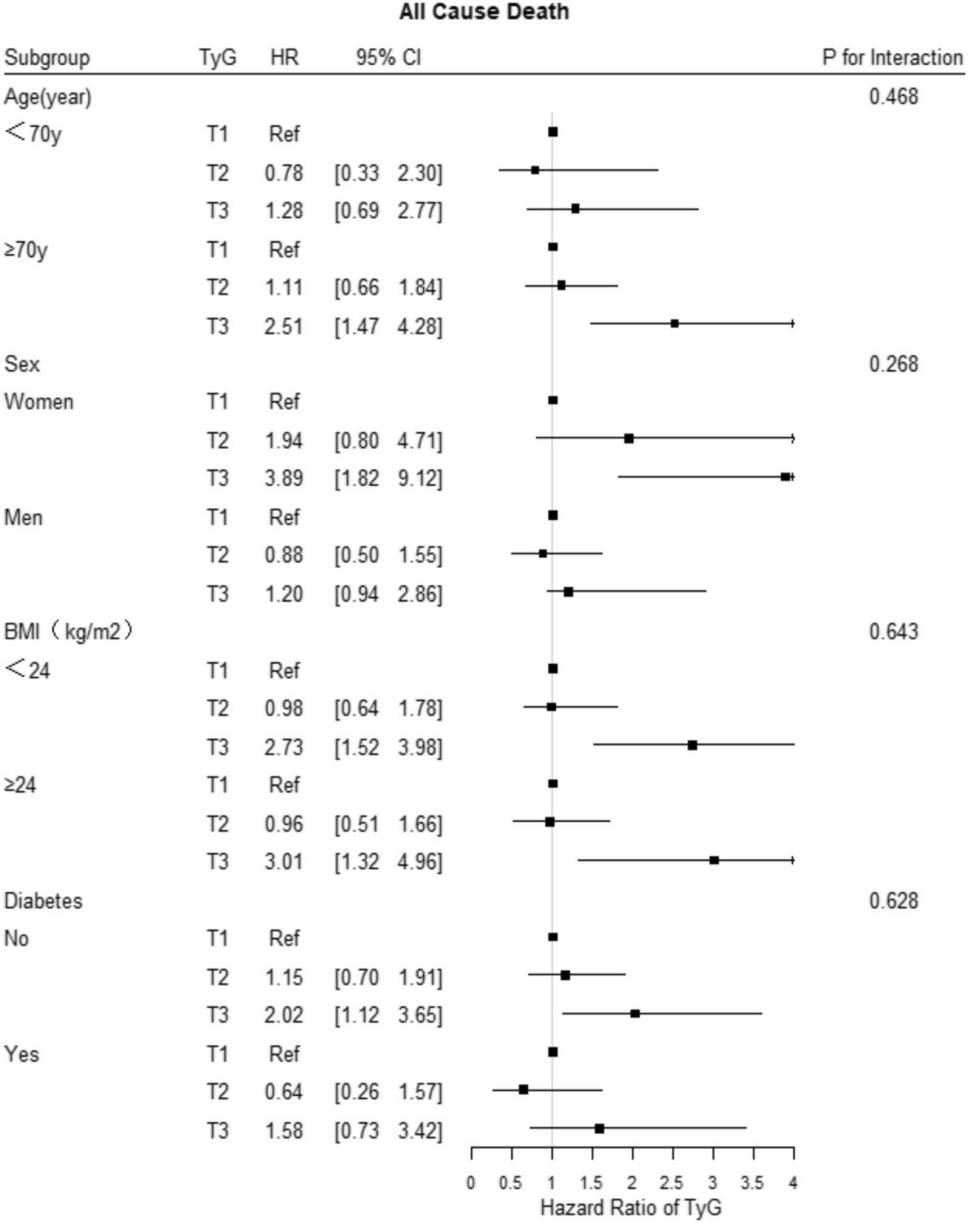
Forest plot of all-cause death according to different subgroups. Adjusted model included age, sex, TyG index, body mass index, systolic blood pressure, diastolic blood pressure, heart rate, HbA1C, C-creative protein, hematocrit, red blood cell distribution width, BNP, sodium, albumin, creatinine, uric acid, ALB, HDL-C, LVEF, history of hypertension, ischemic cardiomyopathy, diabetes mellitus, valvular heart disease, atrial fibrillation, hyperlipidemia, and statin, aldosterone antagonist, digoxin, diuretic, β-blocker, antiplatelet agent, ACEI/ARB/ARNI, insulin, sodium-glucose contrasporter-2 inhibitor, and metformin uses

**Fig. 6:**
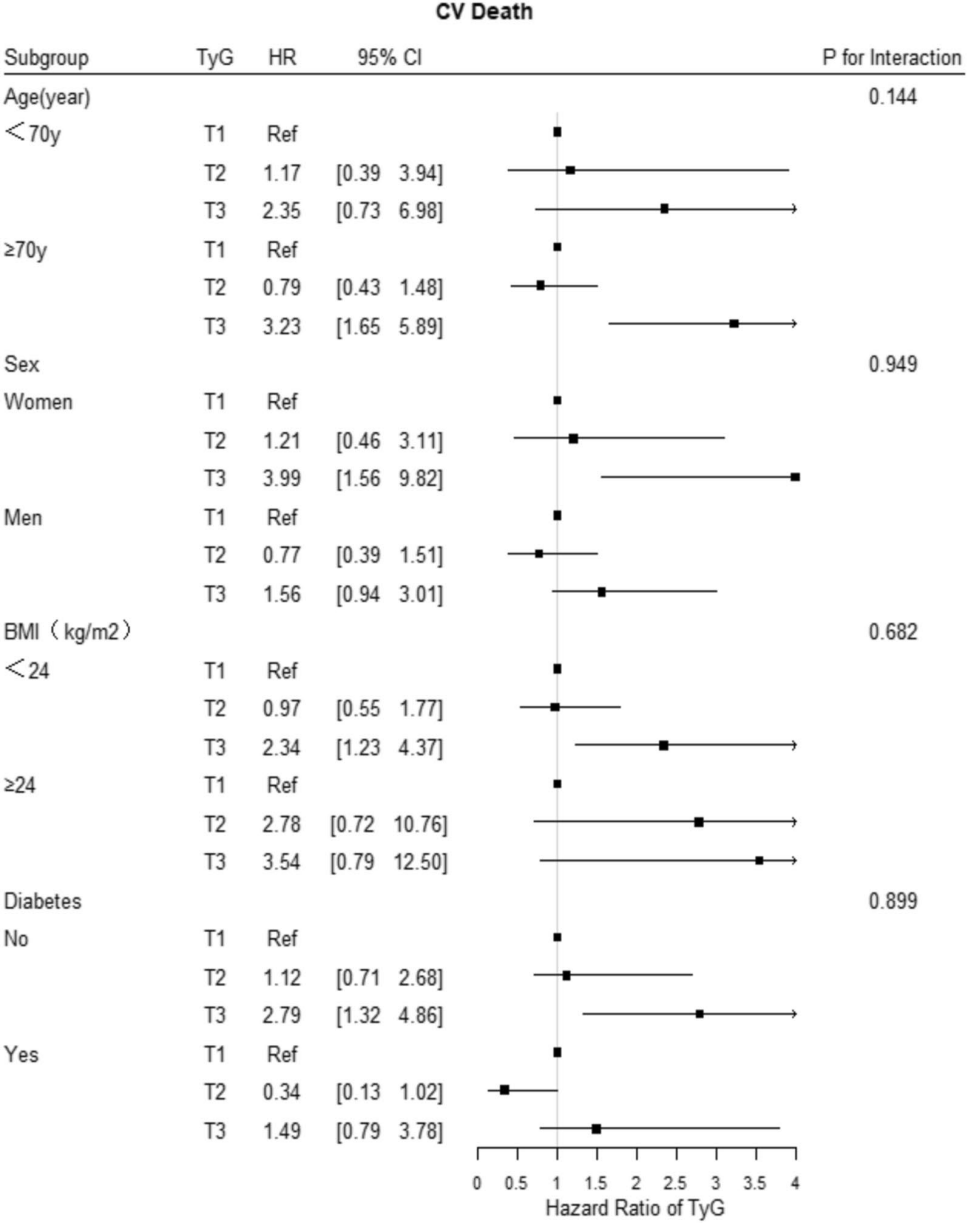
Forest plot of CV death according to different subgroups. Adjusted model included age, sex, TyG index, body mass index, systolic blood pressure, diastolic blood pressure, heart rate, HbA1C, C-creative protein, hematocrit, red blood cell distribution width, BNP, sodium, albumin, creatinine, uric acid, ALB, HDL-C, LVEF, history of hypertension, ischemic cardiomyopathy, diabetes mellitus, valvular heart disease, atrial fibrillation, hyperlipidemia, and statin, aldosterone antagonist, digoxin, diuretic, β-blocker, antiplatelet agent, ACEI/ARB/ARNI, insulin, sodium-glucose contrasporter-2 inhibitor, and metformin uses

**Fig. 7:**
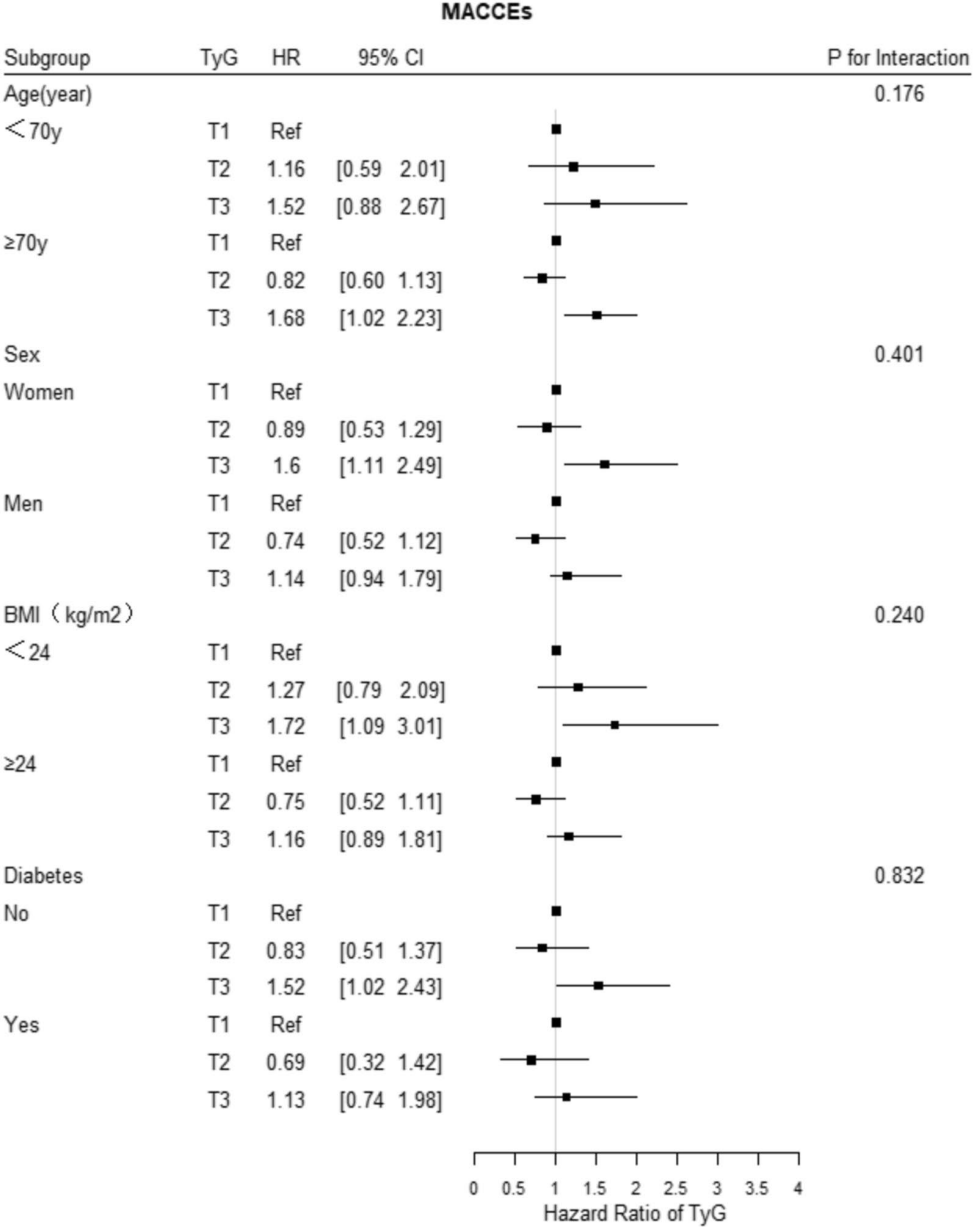
Forest plot of MACCEs according to different subgroups. Adjusted model included age, sex, TyG index, body mass index, systolic blood pressure, diastolic blood pressure, heart rate, HbA1C, C-creative protein, hematocrit, red blood cell distribution width, BNP, sodium, albumin, creatinine, uric acid, ALB, HDL-C, LVEF, history of hypertension, ischemic cardiomyopathy, diabetes mellitus, valvular heart disease, atrial fibrillation, hyperlipidemia, and statin, aldosterone antagonist, digoxin, diuretic, β-blocker, antiplatelet agent, ACEI/ARB/ARNI, insulin, sodium-glucose contrasporter-2 inhibitor, and metformin uses

### Subgroup analysis

The subgroup analysis showed that the associations of the TyG index tertile with the risk of the three primary outcomes were consistent across the subgroups, including age, sex, BMI, LVEF, and history of diabetes mellitus (Figs. 5, 6, 7). In addition, the more significant trends of the TyG-index were observed among females for the incidence rates of all-cause death and cardiovascular death. Meanwhile, there were no interactions between the TyG index and the variables in subgroup analyses (all p values for interaction > 0.05).

## Discussion

In this retrospective research, we first demonstrated the associations of the TyG index with the risk of all-cause death, CV death, and MACCEs (including non-fatal MI, non-fatal stroke, and rehospitalization due to HF) in patients with ADHF. We found that patients with elevated TyG index were at greater risk of adverse endpoints, regardless of their diabetic status. The results indicated that the TyG index was an independent predictor of adverse events in ADHF patients. Most significantly, this research proposed a simple and efficient method for evaluating IR to optimize risk stratification of mortality and cardiovascular disease recurrence in ADHF patients.

### Insulin resistance, TyG index, and CVD risk

Epidemiological studies have shown that ADHF is a prevalent and serious condition with significant morbidity and mortality, imposing a growing global public health burden [33]. We urgently need to explore novel biomarkers, mechanisms, and targeted measures in ADHF. Previous studies have shown that IR is prevalent in patients with HF, and precedes the development of heart failure [34]. IR is also an indicator of heart failure and heart function deterioration [35, 36].

IR is defined as a clinical or experimental condition in which glucose uptake and utilization are impaired [37]. According to previous studies, IR is associated with cardiovascular risk in different populations by inducing imbalances in glucose and lipid metabolism, and triggering oxidative stress and inflammatory response, endothelial dysfunction, and ectopic lipid accumulation [15, 37–39]. HOMA-IR is a comparatively extensive method for IR assessment [14]. The TyG index is strongly correlated with HOMA-IR and the hyperinsulinemic-euglycemic clamp (HIEC), even outperforming the HOMA-IR [18, 19].

In recent years, numerous clinical studies have shown that the TyG index is correlated with the risk of incident cardiovascular disease. Two retrospective cohort studies by Sánchez Iñigo et al. and Li et al. showed that healthy participants with elevated TyG index are at higher risk of cardiovascular events [21, 24]. Another cross-sectional observational study by Thai et al. reported that elevated TyG index is correlated with the incidence and severity of coronary stenosis in patients with type 2 DM [23]. Park et al. reported that the TyG index is an independent predictor of the progression of coronary artery calcification [40]. Liu et al. also reported a high TyG index increase the risk of subclinical myocardial injury [41]. Two studies showed that patients with elevated TyG index are more likely to develop hypertension [22, 42], which was also confirmed in the present research. Moreover, Lu et al. found that in a non-diabetic population, higher TyG index was significantly associated with subclinical atherosclerosis in women compared to men [43]. Further researches have shown that the TyG index is more substantially associated with arterial stiffness, coronary artery calcification, and carotid atherosclerosis than HOMA-IR [17, 20, 44]. In addition, several studies proposed that the TyG index can also predict the risk of major adverse cardiac events in patients with acute coronary syndrome (ACS) and DM [25, 26]. Hu et al. indicated that the TyG index was a better predictor of cardiovascular risk than FPG or HbA1C in ACS patients undergoing percutaneous coronary intervention (PCI) [27].

### Association and potential mechanisms between TyG index and ADHF

A retrospective cohort study reported the TyG index is correlated with the prognosis of patients with CHF and type 2 DM [45]. Another study of 132 hospitalized HF patients revealed the TyG index was a novel biomarker of myocardial fibrosis and a valuable risk stratification metric in HF [46]. Our results in the present study are consistent with these observations. Nevertheless, the predictive value of the TyG index in ADHF is not fully recognized. In this study, we investigated the associations of the TyG index with clinical outcomes in patients with ADHF. The mechanisms underlying the association of the TyG index with adverse prognosis of heart failure may include the following. First, IR can induce changes in substrate metabolism and inefficient energy metabolism, thereby hampering the normal myocardial response to injury [11, 34]. In addition, the metabolic efficiency of the myocardium is further dampened in ADHF patients due to the downregulation of genes regulating the beta-oxidation of fatty acids [47, 48]. Secondly, IR contributes to subcellular component abnormalities, including lipotoxicity, oxidative stress, mitochondrial dysfunction, endoplasmic reticulum (ER) stress and impaired calcium signaling [13, 34, 47, 48]. Thirdly, IR promotes inappropriate activation of the sympathetic nervous system and renin–angiotensin–aldosterone system [13, 49]. Fourthly, IR is correlated with hyperglycemia and free fatty acid elevation, inducing immune cells infiltration in adipose tissue and macrophage activation [13]. The secretion of pro-inflammatory mediators by immune cells and macrophage leads to local and systemic low-grade inflammation [13, 47, 50]. As the disease progresses, the vicious cycle of the above conditions contributes to cardiomyocyte injury and death, cardiac hypertrophy and cardiac fibrosis [13, 51]. At the same time, heart failure also contributes to insulin resistance, which in turn leads to further deterioration of heart function [52, 53].

Therefore, for optimizing the risk stratification in patients with heart failure and exploring more potential therapeutic options, it is necessary to examine the link between HF and IR. The TyG index is more convenient for routine screening in clinical practice than HOMA-IR. The TyG index could be monitored in patients with HF, particularly ADHF cases because of difficulty in treatment and poor prognosis.

### Study limitations

There were several limitations in this study. Firstly, due to the limitations of a retrospective design, we were unable to dynamically measure TyG in patients over the follow-up period. Secondly, as this was a single-center study with a limited sample size, data bias could not be avoided despite correction for multiple confounding factors. Thirdly, because of the limited clinical information available, the differences between TyG and other IR indicators, including HOMA-IR and HIEC, in the prognosis of ADHF were not studied. Furthermore, prospective cohort studies are required to validate our findings.

## Conclusion

In summary, this study demonstrated that the TyG index was directly correlated with poor prognosis in patients with ADHF regardless of DM status. Elevated TyG index could be a predictor and risk stratification tool for all-cause death, cardiovascular death, and MACCEs in patients with ADHF.

## Data Availability

The information and data of the study population were extracted from Hospital Information System. The datasets are not publicly available because the individual privacy of the participants should be protected. Data are however available from the corresponding author on reasonable request.

## Abbreviations

ADHF: Acute decompensated heart failure
CHF: Chronic heart failure
IR: Insulin resistance
CV: Cardiovascular
HOMA: The homeostasis model assessment
TyG: Triglyceride‑glucose
FPG: Fasting plasma glucose
TGs: Triglyceride levels
BMI: Body mass index
SBP: Systolic blood pressure
DBP: Diastolic blood pressure
DM: Diabetes mellitus
TC: Total cholesterol
LDL-C: Low-density lipoprotein cholesterol
HDL‑C: High‑density lipoprotein cholesterol
RDW: Red blood cell distribution width
CRP: C-creative protein
BNP: B-type natriuretic peptide
ALB: Albumin
WBC: White blood cell
ALT: Aspartate transaminase
AST: Alanine aminotransferase
MI: Myocardial infarction
MACCEs: Major adverse cardiac and cerebral events
SD: Standard deviation
IQR: Interquartile range
ANOVA: Analysis of variance
IVSTD: Interventricular septal thickness at diastole
LVPWTD: Left ventricular posterior wall end-diastolic thickness
LVDD: Left ventricular end-diastolic diameter
LAD: Left atrial diameter
LVEF: Left ventricular ejection fraction
ACEI: Angiotensin-converting enzyme inhibitor
ARB: Angiotensin receptor inhibitor
ARNI: Angiotensin receptor neprilysin inhibitor
SGLT2i: Sodium-glucose contrasporter-2 inhibitor
HR: Hazard ratio
CIs: 95% Confidence intervals
IMD: Ischemic cardiomyopathy
HIEC: Hyperinsulinemic-euglycemic clamp
PCI: Percutaneous coronary intervention
ACS: Acute coronary syndrome
ER: Endoplasmic reticulum
